# A Clinical Study Investigating Whether the Tongue-Out Position Improves the Quality of the Anatomical Appearance of the Pharynx on CT Imaging

**DOI:** 10.3389/fsurg.2021.732607

**Published:** 2021-09-29

**Authors:** Jin-Long Wu, Rui-Gang Ge, Guang-Jian Tang

**Affiliations:** ^1^Department of Medical Imaging, The Third People's Hospital of Datong, Shanxi, China; ^2^Department of Radiation Oncology, The First Medical Center of People's Liberation Army General Hospital, Beijing, China; ^3^Department of Radiology, The First Hospital of Peking University, Beijing, China

**Keywords:** tongue-out position, pharynx anatomy, CT, swallowing artifact, head and neck

## Abstract

**Objective:** To evaluate the effect of using the tongue-out position on the quality of the anatomical appearance of the pharynx on computed tomography (CT) images.

**Methods:** The data from enhanced CT thin-section images of the head and neck in 119 cases scanned were retrospectively analyzed. The cases were divided into two groups based on the position of the tip of the tongue on the images: the tongue-out group (63 cases) and non-tongue-out group (56 cases). Two observers separately evaluated the anatomy of the soft palate, uvula, palatine tonsils, epiglottis, epiglottic fossa, pyriform fossa, arytenoid folds, and tongue on all images. The Kappa test was applied to assess the consistency of scores between the two observers. In the case of data that satisfied the normal distribution, the significance of the difference in the average scores between the two groups was tested using an independent samples *t*-test with a value of *p* > 0.05. In the case of data that did not satisfy the normal distribution, the Mann–Whitney *U* test was adopted to test the significance of the difference in the average scores between the two groups using a value of *p* < 0.05. The number of cases with swallowing artifacts on the CT images in both groups was statistically analyzed and the chi-square test was used to determine whether the difference in the incidence of artifacts between the two groups was significant.

**Results:** The Kappa test showed good consistency between the two observers scoring of the soft palate, uvula, epiglottis, epiglottic fossa, pyriform fossa, aryepiglottic folds, and tongue. The image scores of the soft palate, uvula, epiglottis, epiglottic fossa, and tongue in the tongue-out group vs. the non-tongue-out group did not satisfy the normal distribution. The Mann–Whitney *U* test showed that the differences in the image scores between the two groups were statistically significant in all cases (*p* < 0.05). The incidence of swallowing artifacts in the tongue-out group and the non-tongue-out group was 15 and 32%, respectively. The result of the chi-square test showed that the difference in the incidence of swallowing artifacts between the two groups was statistically significant (*p* = 0.037).

**Conclusion:** The tongue-out position facilitated an improvement in the CT appearance of pharyngeal anatomy and was associated with a reduction in the incidence of swallowing artifacts.

## Introduction

Computed tomography (CT) of the head and neck is an important diagnostic imaging method in the investigation of the many diseases that affect the pharynx and larynx. CT has a high spatial resolution and can demonstrate the complex anatomy of the laryngopharynx to detect lesions and has the unique advantage of being able to demonstrate the relationship between lesions and normal anatomical structures. However, the anatomical structure of the neck is complex with poor tissue density. In addition, when CT imaging is performed in the supine position, the falling back of the tongue root narrows the air-containing cavity of the oropharynx and laryngopharynx, affecting the identification of anatomical abnormalities.

In clinical practice, we had observed that when patients were asked to adopt the tongue-out position during head and neck CT examinations, the anatomical appearance of the oropharynx and laryngopharynx was improved, and to a certain extent, the swallowing movements of the patients during the examination were inhibited. A search of the literature failed to reveal any relevant reports, and no descriptions of tongue-out position in CT and magnetic resonance imaging (MRI) scanning methods of the pharynx were found in the radiology texts (1, 2). Therefore, this study was designed to evaluate the value and clinical significance of using the tongue-out position during head and neck CT examinations.

## Methods and Materials

### Patient Data

The image data of patients who had undergone head and neck CT examinations between January 2017 and December 2018 were retrospectively retrieved from the database of the Third People's Hospital of Datong. The inclusion criteria were as follows: patients who underwent head and neck plain and enhanced CT scans with complete image data, including those with enhanced venous phase retrospective thin-layer reconstruction. The exclusion criteria were as follows: (1) patients with oropharyngeal and laryngopharyngeal tumors with obvious occupying effecting and poorly displayed pharyngeal anatomy, (2) patients with dentures and other metallic foreign bodies resulting in substantial artifact interference, (3) patients with impaired consciousness who were unable to cooperate with the examination, and (4) patients who did not undergo retrospective reconstruction with 1.25 mm layer thickness.

**Figure 1:**
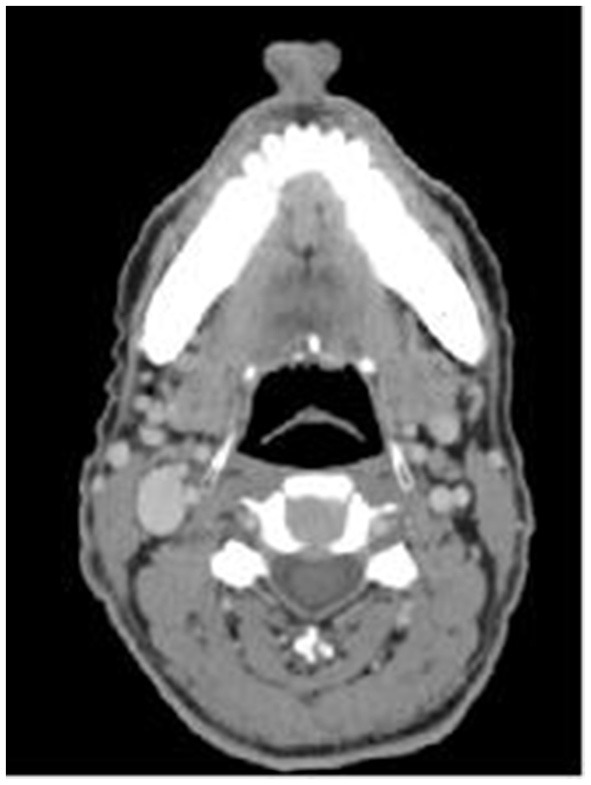
The score of 2 points, with symmetrical bilateral epiglottic fossas and smooth edges.

In total, 119 cases were enrolled in the study. The tongue-out position was defined as the tip of the tongue >1 cm anterior to the incisors based on the CT images (Figure 1). This definition was used to divide the enrolled patients into two groups: There were 63 cases in the tongue-out group and 56 cases in the non-tongue-out group.

### Apparatus and Methods

All studies were acquired with one of two CT scanners: the GE Bright-speed 64 VCT or the Philips Brilliance 256 iCT. Patients were placed in a supine, head-in-first position and the scanning area extended from the base of the anterior cranial fossa to the level of the aortic arch.

The patient was asked to breathe calmly without holding their breath prior to scanning and was advised to avoid swallowing during scanning. In the tongue-out group, prior to scanning, the patient was trained to extend the tongue as far out of the mouth as possible and to gently bite down with the incisors during scanning. The patients gave written informed consent prior to the examination having been informed of the importance of contrast and its risks.

The scanning parameters were as follows: (1) Tube voltage of 120 KV, with automatic tube current with an upper limit of 400 mA, (2) a noise index of 10, (3) collimation of 0.625 mm × 64 or 0.75 mm × 128 with a reconstruction layer thickness of 5 mm and a layer spacing of 0 mm, (4) scanning data during the enhanced venous phase were retrospectively reconstructed with a layer thickness of 1.25 mm, a field of view of 194, and a matrix of 512 × 512, (5) for enhancement, using automatic trigger technology, 90 mL of the contrast agent Ultravist (300 mgI/mL) was injected at an injection rate of 3.5 mL/sec, and (6) the region of interest was placed on the side of the descending aortic arch, the trigger threshold was 120 HU, the pulse phase scan was performed after the trigger, and the venous phase scan was performed at an interval of 35 s following the end of scanning.

### Image Analysis

Two imaging physicians, each with more than 5 years of experience in reporting head and neck images, independently analyzed image scores on PACS (picture archiving and communication systems).

**Figure 2:**
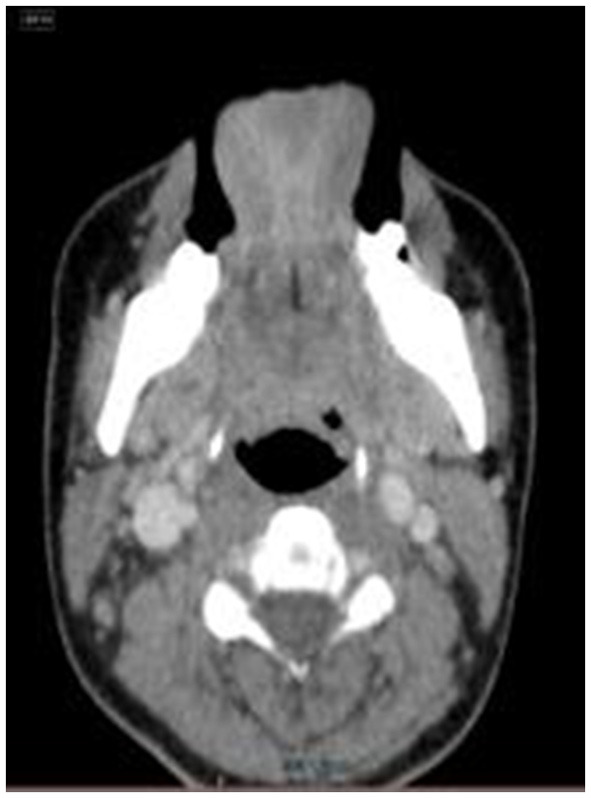
The score of 1 point,with significantly narrowed bilateral epiglottic fossas and partially closed.

**Figure 3:**
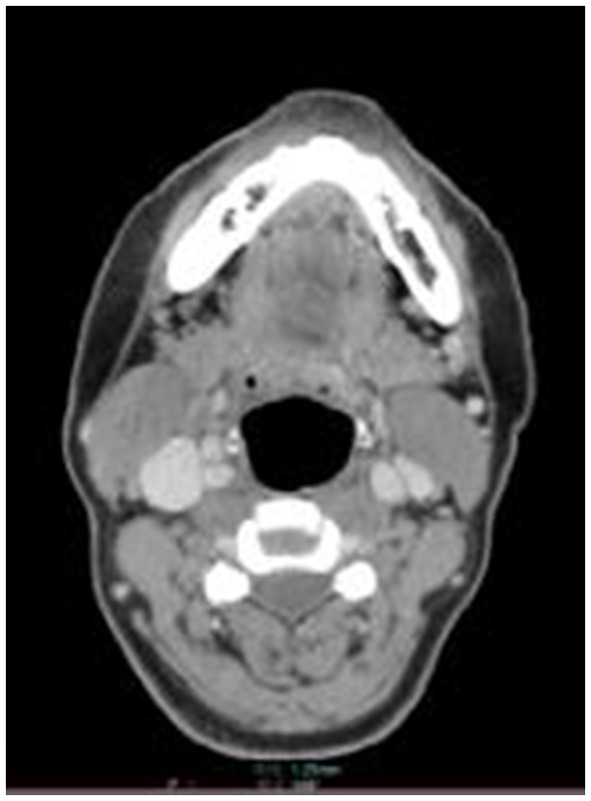
The score of 0 point,with closed bilateral epiglottic fossas and poor demarcation with adjacent soft tissues.

**Figure 4:**
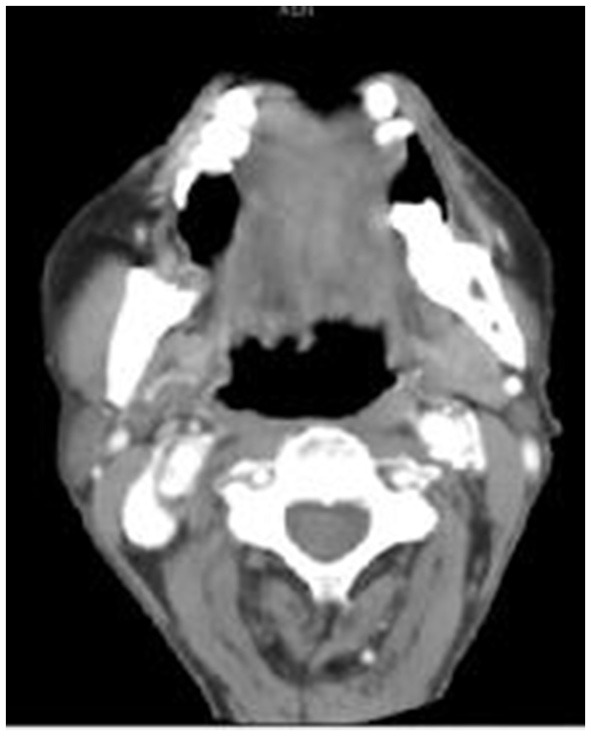
Swallowing artifacts: In the tongue-out group, the swallowing artifacts were visible at the tongue root, with no artifacts in the cervical spine.

**Figure 5:**
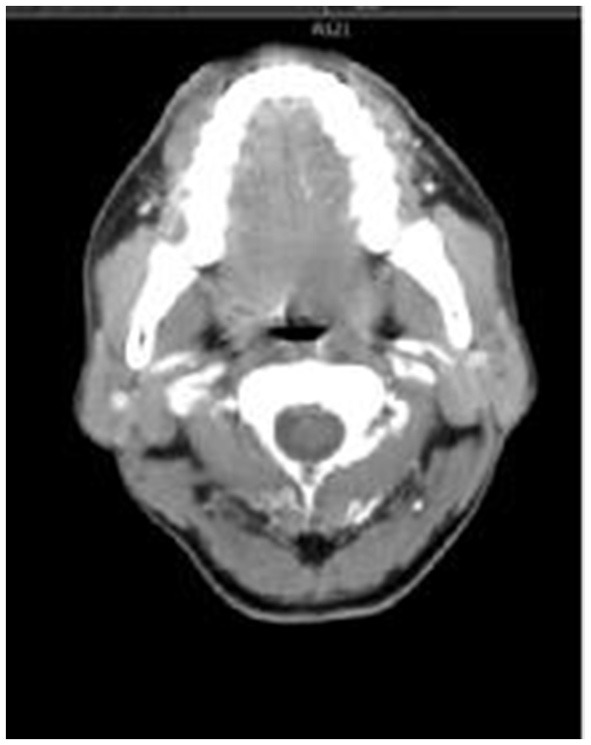
Swallowing artifacts: In the non-tongue-out group, swallowing artifacts were visible in the bilateral palatine tonsils and tongue root, and there were no artifacts in the cervical spine.

(1) The retrospectively reconstructed images in the enhanced venous phase thin-layer transverse axial position were selected to evaluate and observe the following anatomical structures: the soft palate, uvula, palatine tonsils, epiglottis, epiglottic fossa, pyriform fossa, arytenoid folds, and tongue. Criteria for the observed indicator scores (Figures 1–3) were as follows: Those with clear anatomy and strong diagnostic confidence were given 2 points, those with recognizable anatomy but poor diagnostic confidence were given 1 point, and those with an unsatisfactory appearance of anatomical structures where a diagnosis was not possible were given 0 points.

(2) The presence of swallowing during the enhanced arterial and venous phases was defined as the existence of motion artifacts in the pharynx together with clear, artifact-free images of the spine at the same level (Figures 4, 5).

### Data Analysis

The enrolled cases were divided into a tongue-out group and a non-tongue-out group based on the images obtained. The display scores of each anatomical structure by two observers were statistically evaluated using the SPSS 22.0 software for data analysis. The Kappa consistency test was used to analyze the consistency of the two observers' scores for each structure, with *K* ≥ 0.75 indicating good consistency, 0.75 > *K* ≥ 0.4 indicating general consistency, and *K* < 0.4 indicating poor consistency between the two observers.

The average scores of the images between the two observers for the soft palate, uvula, palatine tonsils, epiglottis, epiglottic fossa, pyriform fossa, arytenoid folds, and tongue were statistically evaluated. The Kolmogorov–Smirnov test was applied to test for a normal distribution. In the case of data that satisfied the normal distribution, the significance of the difference in the average scores between the two groups was tested using an independent samples *t*-test, with a value of *p* < 0.05 considered to be statistically significant. In the case of data that did not satisfy the normal distribution, the Mann–Whitney *U* test was adopted to test the significance of the difference in the average scores between the two groups, with a value of *p* < 0.05 considered to be statistically significant.

The difference in the number of cases with swallowing artifacts on CT images between the two groups was statistically analyzed using the chi-square test, with a value of *p* < 0.05 considered to be statistically significant.

## Results

Head and neck CT examinations from a total of 119 cases were included in this study: 76 patients were male, and 43 were female. The age range was 12–82 years with an average age of 50 ± 16 years. Among them, 62 cases were examined for pharyngeal diseases. In the tongue-out group, there were 63 cases with an age range of 14–80 years and an average age of 53 ± 14 years. In the non-tongue-out group, there were 56 cases with an age range of 12–82 years and an average age of 47 ± 18 years. There was no significant difference in age between the two groups (*t* = 1.907, *p* > 0.05). The CT images identified swallowing artifacts in 28 cases.

### Consistency Analysis of the Scores Between the Two Observers

The consistency of the scores between the two observers relating to the images of the soft palate, uvula, epiglottis, epiglottic fossa, pyriform fossa, arytenoid folds, and tongue was good, while the consistency of the scores relating to the palatine tonsils was poor (Table 1).

**Table 1:** Consistency test of the scores between the two observers.

	**Soft palate**	**Uvula**	**Palatine tonsils**	**Epiglottis**	**Epiglottic fossa**	**Pyriform fossa**	**Arytenoid folds**	**Tongue**
Kappa	0.785	0.755	0.227	0.848	0.932	0.825	0.780	0.936

### Comparison of Anatomical Structures on CT in the Tongue-Out and Non-tongue-out Positions

The results of the anatomical structure scores of the pharynx between the two observers are shown in Tables 2, 3. The Kolmogorov–Smirnov test was applied to the image scores in relation to the soft palate, uvula, epiglottis, epiglottic fossa, pyriform fossa, arytenoid folds, and tongue: The test identified that the data did not satisfy the normal distribution (*p* < 0.05, Table 2) and the Mann–Whitney *U* test was adopted to analyze the differences between the two groups. The results showed statistically significant differences in the image scores between the tongue-out group and the non-tongue-out group in relation to the soft palate, uvula, epiglottis, epiglottic fossa, and tongue (*p* < 0.05). However, there were no statistically significant differences in the image scores between the two groups in relation to the pyriform fossa and the arytenoid folds (*p* > 0.05, Table 3).

**Table 2:** The anatomy of the pharynx between the tongue-out group and the non-tongue-out group (The Kolmogorov-Smirnov test).

	**Soft palate**	**Uvula**	**Epiglottis**	**Epiglottic fossa**	**Pyriform fossa**	**Arytenoid folds**	**Tongue**
Kolmogorov-Smirnov Z	3.932	5.518	4.821	5.058	4.433	4.379	3.627
*P*	0.000	0.000	0.000	0.000	0.000	0.000	0.000

**Table 3:** The scores of the anatomy of the pharynx between the tongue-out group and the non-tongue-out group (The Mann-Whitney *U* test).

	**Cases**	**Soft palate**	**Uvula**	**Epiglottis**	**Epiglottic fossa**	**Pyriform fossa**	**Arytenoid folds**	**Tongue**
Tongue-out group	63	1.92 ± 0.37	1.90 ± 0.39	1.81 ± 0.43	1.90 ± 0.39	1.57 ± 0.68	1.56 ± 0.74	1.95 ± 0.28
Non-tongue-out group	56	1.18 ± 0.47	1.61 ± 0.68	1.57 ± 0.49	1.54 ± 0.57	1.57 ± 0.62	1.54 ± 0.63	1.00 ± 0.19
Mann-Whitney *U*		208.00	1228.50	1328.00	1140.00	1724.00	1664.00	195.50
*P*		0.000	0.000	0.003	0.000	0.798	0.526	0.000

### Statistical Analysis of Swallowing Artifacts Between the Tongue-Out Group and the Non-tongue-out Group

In the tongue-out group, swallowing artifacts were identified in 10 cases (15%) on the CT images and were absent in 53 cases. In the non-tongue-out group, swallowing artifacts were identified in 18 cases (32%) on CT images and were absent in 38 cases. The difference in the number of cases with swallowing artifacts between the two groups was statistically significant (*p* = 0.037, Table 4).

**Table 4:** The incidence of swallowing artifact between the tongue-out group and the non-tongue-out group (The chi-square test).

	**Swallowing artifacts (%)**	**Non swallowing artifacts (%)**	**Total**
Tongue-out group	10(15%)	53(85%)	63
Non-tongue-out group	18(32%)	38(68%)	56
Total	28	91	119
			*χ^2^* = 4.36*, P* = 0.037

## Discussion

Clinically, the anatomy of the pharynx is complex, and common disease examination methods include endoscopy, CT, and magnetic resonance imaging (MRI). Endoscopy is an invasive examination and is dependent on the skill of the operator and the location and size of the lesion. Although a biopsy of the lesion can be performed, endoscopy cannot display the deeper parts of the lesion. MRI has been widely popularized and has the advantage of high soft tissue density resolution. However, disadvantages of MRI include the long scanning duration, the relatively obvious breathing and swallowing artifacts, and the relatively low spatial resolution. Moreover, stents and other metal foreign bodies in some patients can impact the use of MRI. Consequently, most hospitals in China still use CT for clinical imaging of the pharynx.

With a short scanning duration, high spatial resolution, and almost no contraindications, enhanced CT examination of the pharynx is widely used in the diagnosis of oropharyngeal, hypopharyngeal, and laryngeal diseases, and is an important diagnostic imaging method for pharyngeal lesions (3–6). The diagnostic value of many post-processing techniques in CT imaging for pharyngeal lesions has been well-reported (7). However, little attention has been paid to the effect of patient position on CT images. The supine position, with the head extended, is often adopted for CT scans of the pharynx (8–10). Due to the falling back of the tongue root in this position, in many cases, the scanned images cannot clearly demonstrate some anatomical structures, and this affects the diagnostic value of the imaging. During scanning, patients are asked to hold their breath or breathe calmly and are also advised that swallowing should be avoided as much as possible. In a study by Liu et al. (11), patients were trained to hold their breath, and scanning during Valsalva breath-holding resulted in a widening of the pyriform fossa and the epiglottis, thus facilitating lesion display. Meng et al. (12) reported that swallowing could be avoided when patients were scanned with the tip of the tongue against the palate. Qi et al. (13, 14) observed the effect of the articulation method on the CT appearances of palatal tumors and normal anatomical structures: When the patient pronounced “ah” during scanning, the oral space increased, and the gap between the tongue and the floor of the mouth also increased. However, a discontinuous articulation or an articulation of inconsistent length negatively affected the scanned and reconstructed images. Although many researchers have proposed various body positions, they have not evaluated the resulting appearance of anatomical structures in-depth or objectively. We observed that the tongue-out position during CT examination of the pharynx improved the appearance of some anatomical structures. Thus, this controlled study was designed to evaluate whether the use of the tongue-out position improved the CT image appearance of the anatomical structures of the pharynx.

The results of this study showed that the CT images of the soft palate, uvula, epiglottis, epiglottic fossa, and tongue were clearer in the tongue-out position and that when compared with the image scores in the non-tongue-out group these differences were statistically significant (**Tables 1–3**). Possible reasons for this may include: (1) The tongue extends out of the oral cavity during the tongue-out position, thus avoiding the falling back of the tongue root. This increases the space around structures such as the epiglottic fossa, epiglottis, soft palate, and uvula, exposing them to the air of the oropharynx and laryngopharyngeal cavity. This in turn results in a clearer shape, widening the space between the tongue and the floor of the mouth, and making tongue lesions easier to identify, (2) the bilateral palatoglossal muscles elongate during the tongue-out position causing the soft palate and the palatal lobe to drop relatively, showing these structures more clearly, (3) contraction of the bilateral palatopharyngeal muscles during the tongue-out position may help to lift the larynx, and (4) during the tongue-out position, the median folds of the lingual epiglottis may pull the root of the epiglottal cartilage, This action, together with the epiglottis moving posteriorly and the epiglottal crypt being enlarged, may improve the appearance of the anatomical structures and the lesion.

In contrast, there were no statistically significant differences between the two groups for the image scores of the pyriform fossa and arytenoid folds. These structures are distant from the tongue, and therefore a tongue-out position may have little effect on the appearances of these anatomical structures on CT.

Motion artifacts, created when the patient swallows during scanning, are another important factor affecting the image quality of pharyngeal CT images. During the enhancement phase of the scan, the intravenous injection of iodine contrast causes a burning sensation in the throat that will induce the patient to swallow. When using the tongue-out position, the tongue muscle is under tension and this has a certain inhibitory effect on swallowing, thus reducing the impact of CT image interference due to swallowing artifacts on the diagnostic value of the images. The results of this study showed that the percentage of cases with swallowing artifacts in the tongue-out group was lower than that in the non-tongue-out group and this difference in incidence was statistically significant.

In this study, in all cases, the CT scans demonstrated normal anatomy of the oropharynx and laryngopharynx (Table 4). The diagnostic value of the tongue-out position on the CT assessment of pharyngeal lesions, therefore, remains unclear. As this study was retrospective, the method, and language used to instruct patients to carry out the tongue-out position before scanning was not standardized. In addition, the degree of tongue-out position obtained relied on patient understanding, and it was, therefore, difficult to achieve consistency. These are all possible limitations of this study.

## Conclusion

Asking the patient to adopt the tongue-out position prior to CT scanning is easily accepted by the patient. This simple maneuver improves the CT appearance of the oropharynx and laryngopharynx and may be conducive to better detection and diagnosis of lesions.

## Data Availability Statement

The original contributions presented in the study are included in the article/supplementary material, further inquiries can be directed to the corresponding authors.

## Ethics Statement

This study involving human participants were reviewed and approved by Ethics Committee of The Third People's Hospital of Datong. The study was a retrospective analysis and patients were therefore not required to give written informed consent.

## Author Contributions

G-JT and J-LW conceived the idea and conceptualized the study and drafted the manuscript. J-LW collected the data. R-GG and J-LW analyzed the data. R-GG reviewed the manuscript. All authors read and approved the final draft.

## Conflict of Interest

The authors declare that the research was conducted in the absence of any commercial or financial relationships that could be construed as a potential conflict of interest.

## Publisher's Note

All claims expressed in this article are solely those of the authors and do not necessarily represent those of their affiliated organizations, or those of the publisher, the editors and the reviewers. Any product that may be evaluated in this article, or claim that may be made by its manufacturer, is not guaranteed or endorsed by the publisher.
